# Divergent Evolutionary Rates of Primate Brain Regions as Revealed by Genomics and Transcriptomics

**DOI:** 10.1093/gbe/evae023

**Published:** 2024-02-05

**Authors:** Xiao-Lin Zhuang, Yong Shao, Chun-Yan Chen, Long Zhou, Yong-Gang Yao, David N Cooper, Guo-Jie Zhang, Wen Wang, Dong-Dong Wu

**Affiliations:** Key Laboratory of Genetic Evolution & Animal Models, Kunming Natural History Museum of Zoology, Kunming Institute of Zoology, Chinese Academy of Sciences, Kunming 650201, China; Kunming College of Life Science, University of the Chinese Academy of Sciences, Kunming 650204, China; Key Laboratory of Genetic Evolution & Animal Models, Kunming Natural History Museum of Zoology, Kunming Institute of Zoology, Chinese Academy of Sciences, Kunming 650201, China; Kunming College of Life Science, University of the Chinese Academy of Sciences, Kunming 650204, China; School of Ecology and Environment, Northwestern Polytechnical University, Xi’an 710072, China; Center of Evolutionary & Organismal Biology, and Women's Hospital at Zhejiang University School of Medicine, Zhejiang University, Hangzhou 310000, China; Liangzhu Laboratory, Zhejiang University Medical Center, Hangzhou 310000, China; Key Laboratory of Genetic Evolution & Animal Models, Kunming Natural History Museum of Zoology, Kunming Institute of Zoology, Chinese Academy of Sciences, Kunming 650201, China; Kunming College of Life Science, University of the Chinese Academy of Sciences, Kunming 650204, China; Key Laboratory of Animal Models and Human Disease Mechanisms of Chinese Academy of Sciences & Yunnan Province, Kunming Institute of Zoology, Chinese Academy of Sciences, Kunming, Yunnan 650201, China; National Resource Center for Non-Human Primates, Kunming Primate Research Center, and National Research Facility for Phenotypic & Genetic Analysis of Model Animals (Primate Facility), Kunming Institute of Zoology, Chinese Academy of Sciences, Kunming, Yunnan 650107, China; KIZ-CUHK Joint Laboratory of Bioresources and Molecular Research in Common Diseases, Kunming Institute of Zoology, Chinese Academy of Sciences, Kunming, Yunnan 650201, China; Institute of Medical Genetics, School of Medicine, Cardiff University, Cardiff CF14 4XN, UK; Center of Evolutionary & Organismal Biology, and Women's Hospital at Zhejiang University School of Medicine, Zhejiang University, Hangzhou 310000, China; Liangzhu Laboratory, Zhejiang University Medical Center, Hangzhou 310000, China; Key Laboratory of Genetic Evolution & Animal Models, Kunming Natural History Museum of Zoology, Kunming Institute of Zoology, Chinese Academy of Sciences, Kunming 650201, China; School of Ecology and Environment, Northwestern Polytechnical University, Xi’an 710072, China; Key Laboratory of Genetic Evolution & Animal Models, Kunming Natural History Museum of Zoology, Kunming Institute of Zoology, Chinese Academy of Sciences, Kunming 650201, China; National Resource Center for Non-Human Primates, Kunming Primate Research Center, and National Research Facility for Phenotypic & Genetic Analysis of Model Animals (Primate Facility), Kunming Institute of Zoology, Chinese Academy of Sciences, Kunming, Yunnan 650107, China; KIZ-CUHK Joint Laboratory of Bioresources and Molecular Research in Common Diseases, Kunming Institute of Zoology, Chinese Academy of Sciences, Kunming, Yunnan 650201, China

**Keywords:** primate, brain, development, evolution, evolutionary rate, transcriptome age

## Abstract

Although the primate brain contains numerous functionally distinct structures that have experienced diverse genetic changes during the course of evolution and development, these changes remain to be explored in detail. Here we utilize two classic metrics from evolutionary biology, the evolutionary rate index (ERI) and the transcriptome age index (TAI), to investigate the evolutionary alterations that have occurred in each area and developmental stage of the primate brain. We observed a higher evolutionary rate for those genes expressed in the non-cortical areas during primate evolution, particularly in human, with the highest rate of evolution being exhibited at brain developmental stages between late infancy and early childhood. Further, the transcriptome age of the non-cortical areas was lower than that of the cerebral cortex, with the youngest age apparent at brain developmental stages between late infancy and early childhood. Our exploration of the evolutionary patterns manifest in each brain area and developmental stage provides important reference points for further research into primate brain evolution.

**Significance**

Our understanding of how various brain regions, particularly non-cortical areas, evolve in primates is incomplete and requires thorough exploration. We performed a comprehensive investigation into the unique evolutionary patterns of each primate brain region, uncovering previously unnoticed evolutionary patterns and identifying potential genetic foundations for primate brain evolution.

## Introduction

Primates, especially humans, are remarkable for their brains, behaviors, and cognitive abilities, unique attributes that have been acquired over an extended period of evolutionary time. Over the last decade, and benefiting from rapid advances in comparative genomics, transcriptomics and epigenomics, studies across primate species have generated many new and fundamental insights into the genetic underpinnings of primate brain evolution (Gilad et al. 2006; Lui et al. 2011; Bernard et al. 2012; Dehay et al. 2015; Bakken et al. 2016; He et al. 2017; Amiri et al. 2018; Kronenberg et al. 2018; Kanton et al. 2019; Agoglia et al. 2021). However, most studies have focused either on the whole brain or the neocortex. Precisely how each specific brain region, and in particular the non-cortical areas, have evolved is not fully understood and remains to be explored in detail. It is therefore very important to acquire a comprehensive understanding of the evolutionary patterns experienced by each individual brain region.

The primate brain comprises a number of functionally distinct structures, the human brain being the most elaborate; different brain structures vary dramatically both within and between primate lineages, reflecting the functional specialization of the brain over evolutionary time (Sousa et al. 2017a; DeCasien and Higham, 2019). There is now abundant molecular genetic evidence to support the notion of functional specialization of the primate brain; for example, in terms of gene expression, different human brain structures exhibit diverse levels of expression with respect to the same groups of genes, and each brain structure has its own uniquely specific gene markers (Hawrylycz et al. 2012; Bhaduri et al. 2021).

Further, previous studies of the entire primate brain have supported the contention that genes involved in brain function evolved more rapidly in primates than in rodents (Dorus et al. 2004), particularly those genes linked to brain development (Dorus et al. 2004). This is reflected in a positive correlation between the ratio of non-synonymous to synonymous mutations of genes and primate brain evolution. Moreover, gene age, namely the estimated time since the emergence of the gene according to phylogenetic analyses, could reflect the evolutionary trajectory of the primate brain (Domazet-Loso and Tautz, 2010). Here, based on previous research (Domazet-Loso et al. 2007; Domazet-Loso and Tautz, 2010; Quint et al. 2012), we leveraged genome sequence data from the Primate Genome Project, which integrated 50 primate genomes sequenced in our laboratory (Shao et al. 2023), utilizing two classic indices—the evolutionary rate index (ERI) and the transcriptome age index (TAI)—to fully investigate the evolution of each brain area in eight major primate ancestral lineages leading to human. Our study not only reveals novel and hitherto underappreciated evolutionary patterns associated with specific primate brain areas but also, and more broadly, identifies the potential genetic underpinnings of primate brain evolution and development.

## Results

### Diverse Evolutionary Rates of Different Brain Areas During Primate Evolution

The functional specialization of primate brain areas implies that different areas might be subject to differing levels of natural selection. This raises basic questions as to which primate brain areas have evolved at a high rate, and which have been relatively conserved over evolutionary time (Lui et al. 2011; Dehay et al. 2015; Sousa et al. 2017b). To address these questions, we deeply investigated the evolutionary rates of various brain regions in primates by integrating massive transcriptome data and selection signals at the gene sequence level. To explore the evolutionary rates, we derived the evolutionary rate index (ERI) by means of the formula ERI = ∑[(dN/dS) ∗ E]/∑E (detailed in Materials and Methods), which was referred to previously (Quint et al. 2012).

**Fig. 1.**
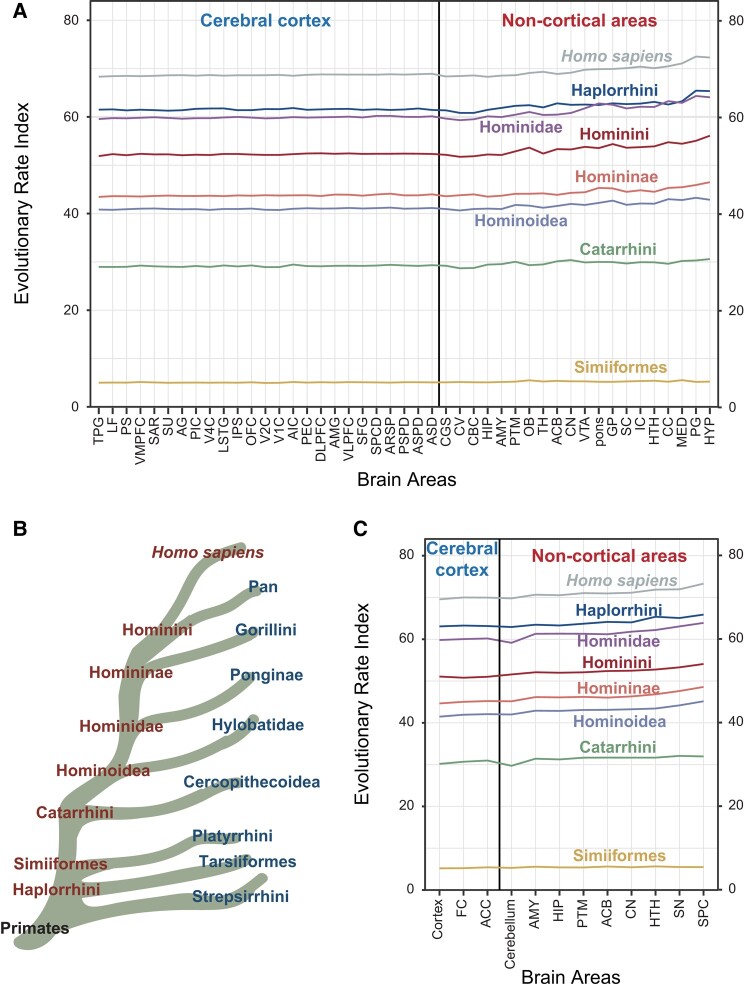
Evolutionary patterns of different brain areas in different primate lineages. (A) Evolutionary rate index of 45 brain areas in different primate lineages based on gene expression data of rhesus macaque brain (Li et al. 2019). The terms ascribed to the brain areas are given below and in supplementary table S1, Supplementary Material online. (B) Simplified diagram of the phylogenies of the different primate lineages. (C) Evolutionary rate index of 12 brain areas in different primate lineages based on human brain gene expression data derived from the Genotype-Tissue Expression (GTEx) project, release V8. The terms ascribed to the various brain areas are given below and in supplementary table S2, Supplementary Material online. CN, caudate nucleus; PTM, putamen; GP, globus pallidus; AMY, amygdala; HIP, hippocampus; CGS, cingulate sulcus; TH, thalamus; HTH, hypothalamus; SC, superior colliculus; IC, inferior colliculus; pons, Pons; CV, cerebellar vermis; HYP, hypophysis; ACB, accumbens nucleus; CC, corpus callosum; VTA, ventral tegmental area; OB, olfactory bulb; MED, medulla; PG, pineal gland; SU, superior postcentral dimple; IPS, intraparietal sulcus; LF, lateral fissure; PEC, parietal area; AMG, anterior marginal gyrus; AG, angular gyrus; LSTG, lateral superior temporal gyrus; TPG, temporal polar gyrus; DLPFC, dorsolateral prefrontal cortex; VLPFC, ventral lateral prefrontal cortex; ASPD, anterior supraprincipal dimple; V1C, primary visual cortex; V2C, visual cortex V2; V4C, visual cortex V4; SFG, superior frontal gyrus; CBC, cerebellar cortex; VMPFC, ventromedial prefrontal cortex; AIC, anterior insula cortex; PIC, posterior insula cortex; OFC, orbitofrontal cortex; PSPD, posterior supraprincipal dimple; SAR, superior arcuate sulcus; PS, principal sulcus; ASD, anterior subcentral dimple; ARSP, arcuate sulcus spur; SPCD, superior precentral dimple; FC, frontal cortex; ACC, anterior cingulate cortex; SN, substantia nigra; SPC, spinal cord.

Employing Primate Genome Project data (Shao et al. 2023), we generated dN/dS (ratio of non-synonymous to synonymous substitutions) values of primate orthologous genes from large scale comparative genomics analyses to reveal the evolutionary genetic patterns exhibited by different brain areas and/or during specific developmental stages. Then, we combined gene expression data from 45 rhesus macaque brain regions (Li et al. 2019) with the dN/dS values of genes from eight major primate ancestral lineages leading to human speciation to generate a measure of the evolutionary rate of each brain area in each primate lineage (Fig. 1A and B, supplementary table S1, Supplementary Material online). Unexpectedly, and contrary to received opinion that contends it was the neocortex which experienced the most rapid evolution in primates (Lui et al. 2011; Dehay et al. 2015), we found that many non-cortical areas, such as the hypophysis (HYP), pineal gland (PG), and medulla (MED), displayed significantly higher evolutionary rates compared to areas of the cerebral cortex (Fig. 1A, Table 1, supplementary table S1, Supplementary Material online); these non-cortical areas are key endocrine glands with the potential to influence individual development, cognitive ability and circadian rhythm (Lovick and Li, 1989; Phansuwan-Pujito et al. 1999; Sapede and Cau, 2013; Alatzoglou et al. 2020; Patel et al. 2020). Moreover, the corpus callosum (CC), hypothalamus (HTH), inferior colliculus (IC), and superior colliculus (SC) also displayed significantly higher evolutionary rates than cerebral cortex (Fig. 1A, Table 1, supplementary table S1, Supplementary Material online); these non-cortical areas are essential for the coordination of activities between the two cerebral hemispheres and for the regulation of both visceral and endocrine activity, as well as the auditory and visual senses (Buckingham, 1977; Wurtz and Albano, 1980; Paul et al. 2007; Trachtman, 2010; Pickles, 2015; Blaauw and Meiners, 2020). Furthermore, combining gene expression data derived from 12 human brain areas from the GTEx project (release V8) (GTEx Consortium, 2020) with the dN/dS values of genes in eight major primate ancestral lineages leading to human speciation as a means to measure the evolutionary rate of each brain area, also yielded similar findings (Fig. 1C, Table 2, supplementary table S2, Supplementary Material online). Notably, *Homo sapiens* and the Haplorrhini lineages exhibited higher evolutionary rates than other primate lineages, suggestive of the rapid evolution of brain areas in these two lineages (Fig. 1A and C, supplementary tables S1 and S2, Supplementary Material online).

**Table 1:** *t*-Test results of ERI values between non-cortical regions and cortical regions, based on rhesus macaque brain expression data (Li et al. 2019)

Lineages	*P* values
*Homo sapiens*	0.0005718
Hominini	0.0001137
Homininae	0.0001537
Hominidae	8.10E−05
Hominoidea	3.21E−05
Catarrhini	1.57E−05
Simiiformes	5.40E−07
Haplorrhini	0.000523

**Table 2:** *t*-Test results of ERI values between non-cortical regions and cortical regions, based on human brain expression data (Zhu et al. 2018)

Lineages	*P* values
*Homo sapiens*	0.001676
Hominini	0.000102
Homininae	0.000831
Hominidae	0.002613
Hominoidea	0.000853
Catarrhini	0.02084
Simiiformes	0.04544
Haplorrhini	0.007484

**Fig. 2.**
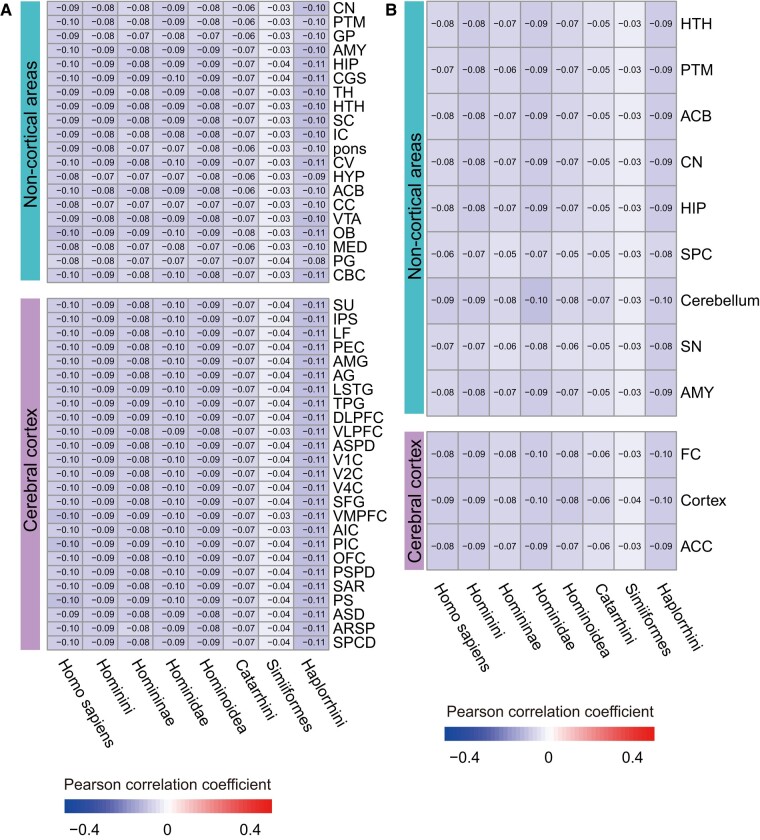
Pearson correlation analyses of different brain areas in different primate lineages. Heatmap displaying the Pearson correlation coefficient between dN/dS in each primate ancestral node and transcriptional abundance in each brain tissue, based on rhesus macaque brain data (A) (Li et al. 2019) and based on the human brain data (B), respectively. Cortical and non-cortical areas are shown separately. The specific Pearson correlation coefficients are shown in the figure. CN, caudate nucleus; PTM, putamen; GP, globus pallidus; AMY, amygdala; HIP, hippocampus; CGS, cingulate sulcus; TH, thalamus; HTH, hypothalamus; SC, superior colliculus; IC, inferior colliculus; pons, Pons; CV, cerebellar vermis; HYP, hypophysis; ACB, accumbens nucleus; CC, corpus callosum; VTA, ventral tegmental area; OB, olfactory bulb; MED, medulla; PG, pineal gland; SU, superior postcentral dimple; IPS, intraparietal sulcus; LF, lateral fissure; PEC, parietal area; AMG, anterior marginal gyrus; AG, angular gyrus; LSTG, lateral superior temporal gyrus; TPG, temporal polar gyrus; DLPFC, dorsolateral prefrontal cortex; VLPFC, ventral lateral prefrontal cortex; ASPD, anterior supraprincipal dimple; V1C, primary visual cortex; V2C, visual cortex V2; V4C, visual cortex V4; SFG, superior frontal gyrus; CBC, cerebellar cortex; VMPFC, ventromedial prefrontal cortex; AIC, anterior insula cortex; PIC, posterior insula cortex; OFC, orbitofrontal cortex; PSPD, posterior supraprincipal dimple; SAR, superior arcuate sulcus; PS, principal sulcus; ASD, anterior subcentral dimple; ARSP, arcuate sulcus spur; SPCD, superior precentral dimple; SPC, spinal cord; SN, substantia nigra; FC, frontal cortex; ACC, anterior cingulate cortex.

Furthermore, to exclude the effect of a strong negative correlation between dN/dS values and transcriptional abundance on the calculation of ERI values, we performed Pearson correlation analyses (Fig. 2A and B). The results showed that, whether they were based on rhesus macaque brain expression profiles (Fig. 2A) or human brain expression profiles (Fig. 2B), the negative correlations between the dN/dS values and transcriptional abundance were weak (−0.1102 to −0.0263), indicating that both dN/dS values and transcriptional abundance could be regarded as complementary measures. In particular, there were only a few differences in the negative correlation coefficients between the cortical areas and non-cortical areas (Fig. 2A and B), indicating that the conclusion that non-cortical areas evolved faster than cortical areas was reliable.

**Fig. 3.**
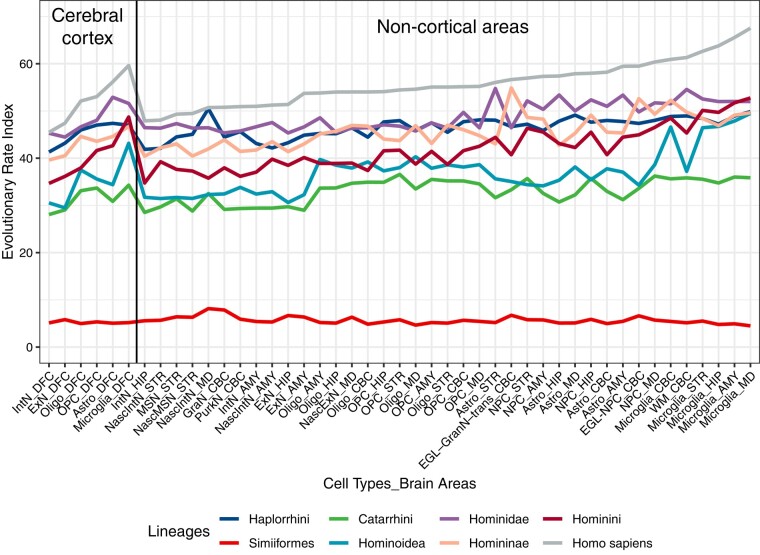
Evolutionary patterns of different cell types of various brain areas in different primate lineages. Evolutionary rate index of 16 cell types of 6 brain areas in different primate lineages based on single cell RNA-seq data of rhesus macaque embryonic brains (Zhu et al. 2018). The terms ascribed to the brain areas are given below. DFC, dorsolateral prefrontal cortex; HIP, hippocampus; AMY, amygdala; STR, striatum; MD, mediodorsal thalamic nucleus; CBC, cerebellum; ExN, excitatory neuron; InN, inhibitory neuron; Astro, astrocyte; OPC, oligodendrocyte progenitor cell; Oligo, oligodendrocyte; NPC, neuronal progenitor cell; NascInN, nascent inhibitory neuron; NascMSN, nascent medium spiny neuron; MSN, medium spiny neuron; NascExN, nascent excitatory neuron; EGL-NPC, external granular layer neuronal progenitor cell; WM-NPC, white matter neuronal progenitor cell; EGL-GraN-trans, external granular layer transition to granule neuron; GraN, granule neuron; PurkN, Purkinje neuron.

Since there were many types of cell in different brain regions, it is necessary to further explore the roles of cell type differences in reconstructing the evolutionary rates. Thus, we utilized the single cell RNA-seq data from six brain regions (including cerebellum, mediodorsal thalamic nucleus, striatum, amygdala, hippocampus, and dorsolateral prefrontal cortex) of two E110 (embryonic day 110) rhesus macaque brains from previously published research (Zhu et al. 2018), combined with dN/dS values from eight major primate ancestral lineages leading to human speciation, to calculate the ERI values of each cell type from each brain region in each primate ancestral node (Fig. 3). The results indicated that many cell types in non-cortical areas displayed higher evolutionary rates compared to that in the dorsolateral prefrontal cortex, especially the microglial cells (Fig. 3). Since microglia are critically involved in many physiological and pathological brain processes, including neurodegeneration (Geirsdottir et al. 2020), the rapid evolution of microglia may suggest their key role in primate brain evolution. These results strongly supported the conclusion that non-cortical areas evolved faster than cortical areas.

The rapid evolution of protein-coding genes related to the primate non-cortical areas including limbic system implies that brain functions related to emotional expression and social cognition have played vital roles during primate evolution. This finding contrasts with the traditional view that the neural basis of emotional behavior has been evolutionarily conserved but is consistent with a previous comparative anatomy study which reported evolutionary specializations of the human limbic system (Barger et al. 2014). Meanwhile, a previous study has also reported that genes expressed in the cortical regions of the brain exhibited lower evolutionary rates than genes expressed in the non-cortical regions (Tuller et al. 2008), a finding which concurred with our conclusions about the evolutionary patterns.

### Rapid Evolution of Primate Brain Development in Early Childhood

**Fig. 4.**
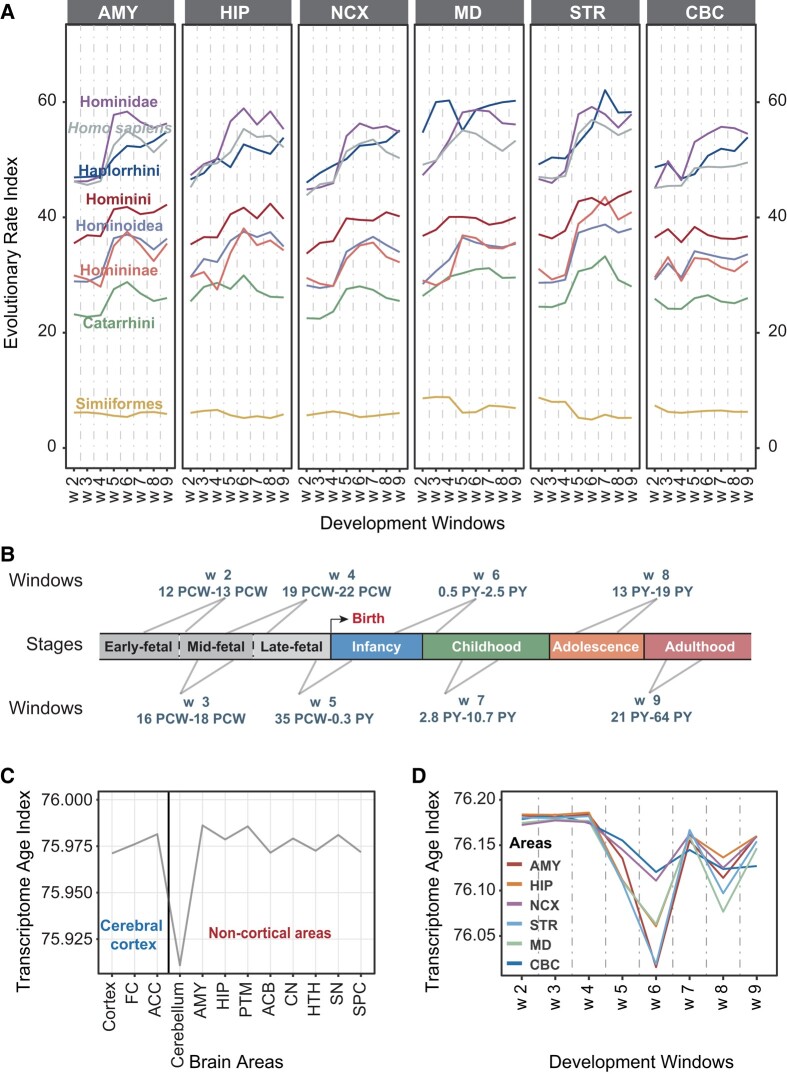
Evolutionary patterns of different developmental stages in different primate lineages and the transcriptome age of different human brain areas in diverse developmental stages. (A) Evolutionary rate index of amygdala (AMY), hippocampus (HIP), neocortex (NCX), mediodorsal nucleus of thalamus (MD), striatum (STR), and cerebellar cortex (CBC) during the course of brain development in different primate lineages based on gene expression data from the developing human brain (Zhu et al. 2018). The division of the developmental windows is as in panel B. (B) The developmental stage spans of the samples used in our research; above and below are the specific developmental time spans of the samples included in each developmental window, referenced from Zhu et al. (2018) and Li et al. (2019). (C) Transcriptome age index of 12 brain areas in human, human brain transcriptomes derived from Aguet et al.; the terms ascribed to the different brain areas are the same as those in Fig. 1C. (D) Transcriptome age index of human amygdala (AMY), neocortex (NCX), hippocampus (HIP), striatum (STR), mediodorsal nucleus of the thalamus (MD), and cerebellar cortex (CBC) in different developmental stages, human brain development transcriptomes were derived from Zhu et al. (2018). The division of the developmental windows was as in panel B.

We further explored whether different brain regions at different developmental stages in various primate lineages might also display diverse evolutionary rates. To this end, we leveraged a total of 577 human brain development transcriptomes from six brain areas, namely cerebellar cortex, mediodorsal nucleus of the thalamus, striatum, amygdala, hippocampus, and neocortex, and covering a range of developmental stages (Zhu et al. 2018), to measure the evolutionary rate of each area in each primate lineage during development (Fig. 4A and B, supplementary table S4, Supplementary Material online).

Five of the six brain areas (the exception being cerebellar cortex) exhibited a similar mountain-like pattern in terms of their evolutionary rates viewed across developmental stages (Fig. 4A). In the amygdala (AMY), hippocampus (HIP), neocortex (NCX), and mediodorsal nucleus of the thalamus (MD), the evolutionary rate trajectory showed a trend to increase from stage w5 (35 pcw [post-conception weeks] to 0.3 py [post-natal years]) to w6 (0.5 py to 2.5 py) (Fig. 4A and B); this stage was also defined as late fetal to early childhood, after which the trajectory flattened out or even declined. In the striatum (STR), the evolutionary rate reached the peak from stage w6 to w7 (2.8 py to 10.7 py) (Fig. 4A and B), which was also defined as childhood. However, in the cerebellar cortex (CBC), the trajectory showed different developmental trends in different primate ancestral nodes (Fig. 4A and B); the evolutionary rate in the Haplorrhini and Hominidae lineages reached its peak from stage w7 to w8 (13 py to 19 py), that was also defined as adolescence, whereas the evolutionary rate in other primate lineages reached its peak from stage w5 to w6. The above results suggested that all brain regions in primates evolved rapidly after birth, and reached their peaks during infancy, childhood, or adolescence (Fig. 4A and B).

The development of primate brain is a long drawn out process that continues well into adulthood. Previous research has shown that early childhood, especially the period between term birth and ∼2 yr of age, is crucial for human brain development and for the establishment of cognitive abilities and behaviors, as well as for influencing the subsequent risk of neuropsychiatric disorders such as autism and schizophrenia (Gilmore et al. 2018). Further, previous research has also indicated that brain development during childhood and adolescence is essential for the maturity of advanced cognitive and behavioral abilities in primates (Pelletier and Neuman, 2014; Vijayakumar et al. 2018). Therefore, the different evolutionary rate trajectories of different brain regions in different primate ancestral nodes have suggested imbalances in primate brain evolution, and these evolutionary patterns may be related to the evolution of specific brain phenotypes in different primate ancestors.

Our findings are consistent with the view that the primate brain has evolved rapidly over the developmental period 0.5 py to 2.5 py (i.e. late infancy to early childhood) (Zhu et al. 2018), which probably accounts for much of the rapid evolution experienced by primates, particularly in the lineages leading to human.

### Varied Transcriptome Ages of Different Human Brain Regions During Development

Based on comparative genome analyses of metazoan genome sequences, a crucial finding has been that a large number of genes have arisen anew during the evolution of the respective lineages (Force et al. 1999; Domazet-Loso and Tautz, 2003; Long et al. 2003; Choi and Kim, 2006). The emergence of these novel genes has contributed greatly to phenotypic evolution, and has also been important for primate brain evolution (Chen et al. 2013).

It is possible to trace evolutionary innovations by calculating transcriptome ages for different human brain areas during development by employing a combination of genome and transcriptome data (Domazet-Loso et al. 2007; Domazet-Loso and Tautz, 2010). Here, relying on the large number of primate genomes recently made available through the Primate Genome Project (Shao et al. 2023), we were able for the first time to ascertain the evolutionary gene age (i.e. the estimated time since the emergence of the gene according to phylogenetic analyses) of human protein-coding genes based on a previous pipeline (Zhang et al. 2011; Shao et al. 2019). The expression data for human protein-coding genes were obtained from previously published work (Zhu et al. 2018; GTEx Consortium 2020). We then applied the genomic phylostratigraphy principle (Domazet-Loso et al. 2007; Domazet-Loso and Tautz, 2010) to calculate the transcriptome age index (TAI) of different human brain areas and different developmental stages to measure the transcriptome age by means of the formula TAI = ∑(A ∗ E)/∑E (detailed in Materials and Methods, Fig. 4C and D, supplementary tables S5 and S6, Supplementary Material online).

We found that the non-cortical areas, especially the cerebellum, tend to exhibit low transcriptome age values and to have accumulated more evolutionarily young genes as compared with the cerebral cortex (Fig. 4C, supplementary table S5, Supplementary Material online), concurring with our above finding that the non-cortical areas evolved more rapidly than the cerebral cortex. Furthermore, consistent with our aforementioned observation on the evolutionary rate trajectory during development, we found that transcriptome age values also displayed a tendency to decrease from developmental stage 0.5 py to 2.5 py (Fig. 4B and D, supplementary table S6, Supplementary Material online). These findings corroborated our earlier conclusion, based on evolutionary rate values, that the stage of development from 0.5 py to 2.5 py evolved particularly rapidly in the lineages leading to human (Fig. 4A, B, and D).

## Discussion

Primate brain evolution has been characterized by a series of distinct genetic changes impacting different brain areas in different primate lineages. It was therefore necessary to analyze multiple brain regions from diverse primate lineages to allow the identification of evolutionary patterns and innovations characteristic of each brain area. We paid particular attention to the non-cortical areas, which have rarely been studied at the genetic level, but which are known to have played key roles during the evolution of the primate brain. In this study, we utilized two classic indices that could be used to define a specific brain area in terms of its evolutionary rate and transcriptome age, with a view to identifying the evolutionary patterns and genetic changes characteristic of each brain area.

The phylostratigraphy approach has been used to trace the evolutionary origin of genes by similarity searches in genomes that represent the entire tree of life. The quantitative formula developed from this approach was the transcriptome age index which combined the phylostratum of a given gene with its expression data at a given developmental stage (Domazet-Loso et al. 2007; Domazet-Loso and Tautz, 2010). Employing the basic principle of this approach, we further utilized two classic formulae to calculate the evolutionary rate index (ERI) (Quint et al. 2012) and transcriptome age index (TAI) (Domazet-Loso et al. 2007; Domazet-Loso and Tautz, 2010), thereby providing an effective means to study evolutionary changes in specific species.

It is well known that the evolutionary rates experienced by different primate brain areas and developmental stages are quite diverse. Indeed, previous research has shown that the cerebral cortex underwent rapid evolution in the primate lineage (Lui et al. 2011; Dehay et al. 2015). However, in this study, we unexpectedly found that the non-cortical areas evolved at an even faster rate than the cerebral cortex. The non-cortical areas constitute an essential part of the brain, responsible for the performance of complex functions and tasks such as emotions, motivation, learning, memory, and other advanced neural activities. The rapid evolution of the non-cortical areas may thus have been closely related to the evolutionary development of primate cognition and behavior. However, the evolution of the primate non-cortical areas has not been closely investigated. Those non-cortical areas that have evolved particularly rapidly, such as the hypophysis and the pineal gland, would clearly be worthy of further study.

Further, our results showed that *H. sapiens* and the Haplorrhini lineages exhibited markedly higher evolutionary rates than other primate lineages, indicating that the brains of *H. sapiens* and the Haplorrhini have undergone rapid evolution. Numerous studies have shown that the human brain evolved rapidly in terms of its relative volume and increased complexity through the expansion of cortical areas and an increase in density of cortical networks (King and Wilson, 1975; Pollard et al. 2006; Prabhakar et al. 2006; Bird et al. 2007; Boyd et al. 2015; Gittelman et al. 2015; Reilly et al. 2015; Dong et al. 2016; Won et al. 2019; Agoglia et al. 2021). What was unexpected was evidencing the rapid evolution of the Haplorrhini brain. However, previous research showed that the relative brain volume increased significantly in the Haplorrhini by comparison with the Strepsirrhini (Shao et al. 2023), which demonstrated that the cerebral cortex of the Haplorrhini brain has expanded rapidly whilst the brain volume of the Haplorrhini has significantly increased during the course of evolution. Our own research has additionally suggested that the Haplorrhini brain experienced rapid evolution, similar to *H. sapiens*.

We also observed that the evolutionary rates of the main brain regions during development display a mountain-like pattern reaching a peak during late infancy to early childhood. Although development continues into adulthood, the developmental stage from late infancy to early childhood is particularly important for the primate brain, since the foundations of adult sensory and perceptual systems which are essential to language, behavior, and emotion are formed at this stage. Our results indicate that the primate brain has experienced rapid evolution in the period from late infancy to early childhood; during this stage, the proliferation and migration of glial precursors, and the differentiation of astrocytes and oligodendrocytes, contribute to the functional maturation of the developing neural circuitry (Li and Barres, 2018), and hence may have promoted the evolution of the primate brain. Furthermore, the transcriptome ages of non-cortical areas were lower than those of the cerebral cortex, and each brain area was at its youngest during late infancy to early childhood, supporting the crucial roles of newly originated primate genes in brain development (Charrier et al. 2012; Dennis et al. 2012; Florio et al. 2015; Ju et al. 2016; Li et al. 2016; Liu et al. 2017; Heide et al. 2020).

In summary, our study reveals uneven evolutionary rates and transcriptome ages across different primate brain regions and developmental stages, indicating that the rewiring programs acting on the gene expression networks in the non-cortical areas and during infancy/early childhood have constituted crucial evolutionary steps toward acquiring our uniquely exceptional brain functions. This study deepens our understanding of brain evolution in primates, and particularly in humans.

## Materials and Methods

### Evolutionary Rate Index (ERI) Formula

To determine the evolutionary rate index (ERI) of different brain regions and developmental stages, we utilized the following formula (Quint et al. 2012):


$$\mathrm{ERI}=\frac{\sum_{i}^{n}{\left(\frac{\mathrm{dN}}{\mathrm{dS}}\right)}_{i}\times E_{i}}{\sum_{i}^{n}E_{i}}$$

where (dN/dS)*_i_* is a value that represents the ratio of non-synonymous to synonymous mutations of a given gene *i* in a specific lineage, *E_i_* is the expression value of gene *i* in a specific brain area, and n is the total number of genes analyzed. This formula was used to determine the evolutionary rate index of a particular brain area in a specific primate lineage. Where a particular tissue or developmental stage is described by a high ERI value, this is indicative of a high evolutionary rate for that tissue or stage. To ensure both the representativeness and accuracy of the results, we calculated the ERI index based on gene expression data from human and rhesus macaque, separately (Fig. 1A and C, supplementary tables S1 and S2, Supplementary Material online). A normalized gene expression matrix for human brain was obtained from the GTEx project (release V8) (GTEx Consortium 2020), which included 2,642 samples representing a total of 12 brain areas. The raw transcriptomes of rhesus macaque brain were obtained from the dataset published by our own laboratory (Li et al. 2019). The 590 transcriptomes representing 45 brain areas were re-mapped to the reference genome (*Macaca mulatta*, Mmul_10, Ensembl v99) using STAR (Dobin et al. 2013) without providing junction annotation; those samples with >10 million uniquely mapped reads, and with the proportion of uniquely mapped reads being greater than 80%, were retained. Finally, the remaining 566 transcriptomes were used for downstream analyses (supplementary table S3, Supplementary Material online). We normalized the expression counts and calculated the TPM (transcript per million) for further analyses. The dN/dS values of 10,279 orthologous genes of *H. sapiens*, Hominini, Homininae, Hominidae, Hominoidea, Catarrhini, Simiiformes, and Haplorrhini were obtained from our laboratory's previously published paper (Shao et al. 2023); the orthologous genes were identified between human (*H. sapiens*, GRCh38), each of the other primate species and the outgroup species, the Chinese tree shrew, based on criteria including reciprocal best blastp hit (RBH), gene synteny and genome synteny (Shao et al. 2023). The evolutionary rate index yielded similar results whether based upon the human or the rhesus macaque brain transcriptome, indicating that our method was robust (Fig. 1A and C, supplementary tables S1 and S2, Supplementary Material online).

In addition, the ERI formula was also used to evaluate the evolutionary rates of different brain areas at different developmental stages. Normalized gene expression values of developing human brains were obtained from the previously published paper (Zhu et al. 2018); we selected 577 transcriptomes from six brain regions employing 8 developmental windows ranging from 12 pcw to 64 py.

### Transcriptome Age Index (TAI) Formula

We evaluated the transcriptome age index (TAI) of different brain areas and developmental stages using the formula (Domazet-Loso and Tautz, 2003; Domazet-Loso et al. 2007):


$$\mathrm{TAI}=\frac{\sum_{i}^{n}A_{i}\times E_{i}}{\sum_{i}^{n}E_{i}}$$

where *A_i_* is a value that represents the evolutionary age of a given gene *i*, i.e. the estimated time since the emergence of that gene according to phylogenetic analysis, *E_i_* is the expression value of gene *i* within a specific brain area, and *n* is the total number of genes analyzed. When a brain area or developmental stage is described by a low TAI value, it indicates enrichment of evolutionarily young genes in that area or at that stage. We dated the origin of human protein-coding genes from the hg38 genome assembly based on the previously described pipelines (Zhang et al. 2011; Shao et al. 2019) from our other paper (Shao et al. 2023). Normalized gene expression values of adult human brains and developing human brains were derived from previously published papers (Zhu et al. 2018; GTEx Consortium 2020). To calculate more reliable ERI and TAI values, we did not set any expression cutoff for the expression data.

## Supplementary Material

evae023_Supplementary_Data

## Data Availability

All the data used in the manuscript were downloaded from previously published studies, as mentioned in the main text. Part of the analysis results were shown in the supplementary tables.
